# Convergence of economic growth and health expenditures in OECD countries: Evidence from non-linear unit root tests

**DOI:** 10.3389/fpubh.2023.1125968

**Published:** 2023-03-17

**Authors:** Esref Ugur Celik, Tolga Omay, Dilaver Tengilimoglu

**Affiliations:** ^1^Department of Economics, School of Business, Atilim University, Ankara, Türkiye; ^2^Department of Business, School of Business, Atilim University, Ankara, Türkiye

**Keywords:** convergence, health expenditure, growth, non-linear unit root tests, OECD

## Abstract

**Introduction:**

The relationship between human capital, health spending, and economic growth is frequently neglected in the literature. However, one of the main determinants of human capital is health expenditures, where human capital is one of the driving forces of growth. Consequently, health expenditures affect growth through this link.

**Methods:**

In the study, these findings have been attempted to be empirically tested. Along this axis, health expenditure per qualified worker was chosen as an indicator of health expenditure, and output per qualified worker was chosen as an indicator of economic growth. The variables were treated with the convergence hypothesis. Due to the non-linear nature of the variables, the convergence hypothesis was carried out with non-linear unit root tests.

**Results:**

The analysis of 22 OECD countries from 1976 to 2020 showed that health expenditure converged for all countries, and there was a significant degree of growth convergence (except for two countries). These findings show that health expenditure convergence has significantly contributed to growth convergence.

**Discussion:**

Policymakers should consider the inclusiveness and effectiveness of health policies while making their economic policies, as health expenditure convergence can significantly impact growth convergence. Further research is needed to understand the mechanisms behind this relationship and identify specific health policies most effective in promoting economic growth.

## 1. Introduction

Health expenditures play an essential role in maintaining economic wellbeing and improving living standards (1). Health expenditure enhances overall wellbeing and prosperity as a form of consumer goods (2). Additionally, increasing the labor productivity resulting from health expenditures further supports the rise of wellbeing.

The impact of human capital on the value added in production through the increase in the quality of the workforce is undeniable. The level of education, both in terms of duration and quality, is generally considered to be the primary factor associated with increased human capital (3). Health expenditures, directly and indirectly, impact access to education and training of the qualified workforce, as they improve living conditions and facilitate healthier participation in the labor market. Health expenditures can also contribute to an increase in productivity by providing a healthier workforce (2).

In addition to its potential positive impact on total factor productivity, increased health spending is anticipated to increase economic output (2, 4). In summary, health expenditures can create healthier and more productive societies. Health expenditures are expected to positively impact economic growth, primarily through the human capital channel. In other words, the fact that health expenditures could be a primary determinant of human capital can significantly impact the output.

Many studies consider human capital as one of the critical determinants of economic growth. The study of Binder and Pesaran (5) also dealt with this relationship. The analysis results in the study show that there are “the same limiting time series properties” between human capital and output. This determination shows that the unit root level of human capital is dominant in determining the unit root level of output. Therefore, it may also mean that human capital convergence leads to output convergence.

On the other hand, considering that health expenditures are one of the main determinants of human capital, an increase in health expenditures means an increase in human capital. Thus, the integration order of the health expenditures series can determine the integration order of human capital. Therefore, based on the impact of human capital on output, it is expected that the integration order of health expenditures will similarly impact the integration order of output. For these reasons, convergence in health expenditures causes convergence in output.

The study's starting point empirically tests the convergence assumption for health expenditures and output mentioned above. The relevant assumption was tested over the variables of health expenditure per qualified worker and output per qualified worker in the 1976–2020 period of 22 OECD countries. The non-linear unit root tests show convergence in health expenditure per qualified worker for all countries included in the analysis. Similarly, almost all the countries in the sample (excluding two countries) show convergence for output per qualified worker. The convergence of health expenditure per qualified worker and output per qualified worker confirms the assumption that the convergence of health expenditure will empirically lead to convergence in output.

Empirical research has important policy implications. The findings suggest that in order to achieve a stable growth structure, countries need to have at least as much spending on healthcare as the average of other countries. This finding is a requirement, but it is not sufficient. It is crucial to transform the nature of human capital to make a significant impact on economic growth. It is also necessary to increase the quality of health expenditures while increasing the value of health expenditures to achieve this efficiency. In other words, the channels for health spending must be properly and effectively prioritized to increase efficiency and contribute to GDP growth.

For this reason, it is of great importance for policymakers to determine appropriate policies consistently when determining the channels through which health expenditures will be transferred. Furthermore, as can be seen from the data used in the study, the data on health spending is facing severe non-linearity (6). This is why considering the non-linearity of health expenditure variables in the analyses performed is vital for the results to be accurate and reliable (6). In addition to all these, another issue that policymakers should pay attention to is the income inequality problem that health expenditures can create. Increasing the quality and quantity of healthcare spending will give individuals access to better education. This qualified education received by healthy individuals will provide high salary expectations.

Consequently, health expenditures should be distributed fairly and equally across all members of society. This situation can manifest itself as both individual and regional differences in income. Therefore, policymakers must inclusively implement health expenditures.

In the first part of the study, a literature review on convergence hypotheses, health expenditure convergence, and growth convergence was conducted. The second part discusses theoretical background. The third part focus on data, methodology, and methods, and an empirical analysis was carried out. In the last part, the study's general findings and the policy proposal are given.

## 2. Literature review

### 2.1. Conceptual framework of the convergence hypothesis

The economics literature classifies the convergence hypothesis into four broad categories. β convergence is one example of this. The income per capita growth in a certain period is estimated using the starting level of income per capita, and time lag is typically used to capture β-convergence. Areas with lower beginning levels of income per capita expand more quickly than regions with greater initial levels of income per capita, according to the regression coefficient of β with a negative sign (7). In the literature, there are two distinct forms of β -convergence. These two types of convergence are unconditional (absolute) and conditional. Absolute β -convergence is founded upon the premise that all nations will eventually reach the same steady state. Therefore, the assumption is that economies are similar in terms of their human capital, savings rates, technology levels, population growth, industrial structures, and various structural characteristics. There is a greater chance of detecting unconditional convergence, while the model is being examined for cross-sections of more homogenous regions in this scenario. As a result, for absolute β convergence, the β parameter is determined without taking a set of control variables into account (8).

Contrarily, each unit will converge to a distinct steady state point when economies have diverse structural characteristics. In this situation, convergence is conditional, and β is calculated by including several structural conditioning elements that are thought to affect the rate of increase in income per capita. Less poor economies may grow slower than wealthier ones, mainly if they are nearer to their steady state, as the rate of convergence is determined by an economy's distance from its steady state (9).

The sigma technique is another alternate method for determining convergence (10). Convergence measures the dispersion of actual income per capita across economies in a region using either the standard deviation or coefficient of variation of the cross-sectional series. Convergence is shown by a decline in the coefficient of variation or standard deviation, demonstrating that disparities income in per capita amongst entities in an area get less over time (11). Divergence occurs when the series' coefficient of variation or standard deviation for income per capita rises with time. When the coefficient of variation or standard deviation alternately rises or falls, a hybrid convergence and divergence occur (11). By regressing time as a variable on the coefficient of variation or the standard deviation of output between nations, one can also examine α-convergence. The presence of α-convergence is established when the parameter of the time variable is both negative and statistically significant, whereas a positive measurement supports divergence. It is demonstrated that nations may not reflect convergence because beta-convergence exists (12). The stochastic convergence technique is another factor to consider when evaluating whether convergence exists (13). The stationarity of income per capita in this scenario leads to the conclusion that convergence exists. Convergence is considered to have occurred when a country's income per capita is stationary compared to that of a reference country. This is because the income's stationarity creates a steady state for the income level (14). Club convergence is a different approach than all other methods (15). Convergence happens in several steady states in this situation. Club convergence is based on the fact that it depends on disparities in a group of nations' income, productivity, or living standards. It seems that nations with comparable income groups tend to converge, making high-income countries more likely to have high expenditures and vice versa.

### 2.2. Health expenditure and growth convergence

Many studies exploring the convergence problem in health expenditures using the concept of convergence have produced mixed results. Following the analysis by Alcalde (16), the convergence of health spending per capita was investigated across 21 OECD countries between the years 1975 and 2003. The study utilized Theil's measure to demonstrate convergence among these nations. The convergence of GDP shares is primarily explained by the convergence of health expenditures, labor productivity, and employment rates. Schmitt (17) states that by using error correction models, it is possible to examine the conditional convergence and β convergence of various categories of social expenditure in 21 OECD countries from 1980 to 2005. The empirical results, taking into account the conditional variables, show that there is significant evidence of convergence in all social expenditure types, particularly in health expenditure. Leiter (18) focuses on the convergence and divergence of healthcare funding, a crucial aspect of any healthcare system. Applying several concepts of convergence, using data from 22 OECD nations between 1970 and 2005, they discover that healthcare finance (HCF) is converged. Fallahi (19) examines the beta and stochastic convergence in total healthcare spending as a percentage of GDP for 11 OECD nations between 1960 and 2006. The findings confirm that stochastic convergence exists for all nations. However, beta convergence is only supported for specific nations before the breakpoints. Panopoulou's (20) study examined convergence in healthcare expenditure per capita among 19 countries during the years 1972–2006 by using the approach of Phillips and Sul. Their findings confirm there is convergence in healthcare expenditure across 17 nations. In the study of Albulescu (21), health expenditures of 6 OECD countries were examined with bound unit root tests for the period 1972–2019. The analysis results indicate the importance of the variety of health systems and the limited convergence process between nations. Kizilkaya and Dag's (22) study used the Fourier unit root test method to analyze the convergence of health expenditures across 17 OECD nations for 1975–2019. The study's findings show that the convergence theory holds in most nations. The non-linear unit root test method was used in Akarsu et al. (23) study to find out if there was a convergence in health spending in 18 OECD nations between 1979 and 2016. The results of this article demonstrate that even though private health expenditures converge, total and public health expenditures per capita diverge.

In order to ascertain whether there was a convergence in health spending in 20 OECD nations between 1971 and 2015, Lee and Tieslau (24) employed the LM unit root testing approach. Convergence among particular country groupings is supported by evidence. In the study by Albulescu et al. (25), bound unit root tests were used to explore the convergence of health expenditures in 6 OECD nations between 1980 and 2012. The ratio of health expenditures to GDP does not appear to be significantly converging. Nghiem and Connelly investigated the convergence of health spending in 21 OECD nations between 1975 and 2014 using Phillips & Sul's technique. According to the findings, there is no indication of convergence in health spending among OECD nations. The LM and RALS-LM unit root tests were used in the study by Payne et al.'s study (26) to analyze the convergence of health expenditures in 19 OECD nations between 1972 and 2008. Health spending per person has converged in most OECD nations.

In the Pekkurnaz (6) research used the non-linear asymmetric heterogeneous panel unit root test, the convergence of health spending in 22 OECD nations between 1980 and 2012 was investigated. The findings do not indicate considerable convergence for all nations; therefore, it would appear most reasonable to consider asymmetry when analyzing convergence regarding health spending. The Lau, Fung, and Pugalis (27) study examined the convergence of health expenditures in 14 OECD countries between 1970 and 2008 using non-linear and panel tests. It is found that there is no convergence in the majority of nations' per capita health expenditures. Using panel data unit root tests, Aslan's (28) study examined the convergence of health spending in 19 OECD nations between 1970 and 2005. The findings of the analysis demonstrated that health spending does not converge across nations. Narayan's (7) study discusses health expenditure convergence for 6 OECD countries for 1960–2000 using LM and IPS unit root tests. The analysis findings indicated that health expenditures in other nations converge on that in the USA.

Considered necessary research has used the idea of convergence to study the issue of growth convergence and has come up with quite a few different results. Uçar and Omay (29) tested the growing convergence of 25 OECD countries in the 1953–2004 period with non-linear unit root tests. Analysis results indicate the existence of income convergence. Furuoka's (30) study tested the income convergence of 5 ASEAN member countries between 1960 and 2015 with Fourier augmented ADF and Fourier ADF tests. The analysis results cannot show income convergence (very low-income convergence is found there). Ceylan and Abiyev's (31) study examined whether there was a convergence in GDP per capita of 15 EU member countries between 1950 and 2015 with non-linear unit root and non-linear asymmetric unit root tests. The analysis gave different results depending on the technique used, and convergence was generally found in very few countries. In the Yaya et al.'s (32) study, growth convergence for 9 Asian countries was tested with the Fourier unit root test with the break, covering the period 1967–2017. The analyses were conducted by classifying them according to the regions and concluded that the income convergence differs according to the regions.

In Lopes and Lopes's (33) study, the income convergence status of 25 countries in the 1950–2016 period was evaluated with the Fourier-type Dickey-Fuller test. The test results reveal that only 10 out of 25 countries have convergence. Holobiuc (34) analyzed the income convergence of 28 EU member states from 2000 to 2018. In the study, the analysis was carried out using α and β convergence techniques. The analysis reveals that different convergence results are encountered in different parts of Europe. Chandra Das et al.'s (35) study focused on growth convergence in BRICS countries. Unlike other studies, the study examined the period of 2006–2017 with quarterly data. Although the panel unit root tests showed conditional convergence in the first period, it was revealed that there was no convergence process in the entire period. Alataş (36) conducted the growth convergence test for 72 countries from 1960 to 2010. Multiple approaches (β, σ, stochastic, and club convergence) were performed within the study. The findings also differed depending on the nature of the test applied.

There are studies that consider the relationship between health expenditures and economic growth–development together. Raghupathi and Raghupathi (2) investigate the relationship between economic performance and public health expenditure in the United States, using data from 2003 to 2014. The results show a strong positive correlation between healthcare expenditure and economic performance measures such as income, output, and labor productivity. However, multi-factor productivity is inversely correlated with healthcare expenditure. The findings suggest that investing in healthcare can improve overall economic performance and calls for further research into universal access to healthcare and its potential benefits. The ARDL method is used in Erçelik's (37) study to analyze the effect of health spending on the output level in Turkey from 1980 to 2015. The study examines the connection between GDP per capita and healthcare spending as a share of GDP. The results of the study suggest that there is a significant long-term relationship between the two variables. Wang's (38) study uses the system generalized method of moments estimation method to investigate the connection between preventive and curative healthcare spending and economic growth in OECD nations. The results suggest that there is a relationship between preventive and curative health spending and economic performance. Wang et al. (39) examine the effect of government health spending on economic development in various regions of China through a non-parametric additive model. The results show that the economic impact of health expenditure is favorable in the western regions and unfavorable in the eastern and central regions. Additionally, the study also demonstrates that there is a strong positive correlation between government health spending and GDP, which has an effect on economic growth across the board and across all regions. Ivankova et al.'s (40) study analyzed the relationship between healthcare financing, treatable mortality of working-age men and women, and economic development in OECD countries Results indicated that healthcare financing negatively impacted treatable mortality in insurance and tax-based health systems and was linked to economic prosperity. The study identified countries with a high potential for health and economic outcomes improvement. Effective interventions should consider regional, social, and economic factors.

## 3. Theoretical background

The convergence hypothesis is a general idea in economics that suggests that, over time, countries tend to converge in terms of their economic performance, such as their growth rate, income per capita, health expenditure, or other variables. The convergence hypothesis is based on the idea that various factors can drive economic growth and development, such as technological advancements, investment in human capital, and access to natural resources. Over time, these factors can help to reduce the economic disparities between countries, leading to convergence in regard to economic performance.

In the case of health expenditure convergence, the hypothesis suggests that, over time, countries will converge in terms of their spending on health as a percentage of their GDP. Various factors, such as advancements in medical technology, increasing demand for health services, and changes in health policy, can drive this convergence.

Overall, the convergence hypothesis suggests that, over time, countries will tend to converge in terms of their performance of the economy, whether in terms of their income per capita, growth rate, health expenditure, or other variables. This hypothesis can be tested using various statistical methods, such as unit root tests or regression analysis, to determine whether convergence is occurring and to identify the factors driving it. The following model is a good base to explain the theoretical connections of the convergence process.

The Neoclassical Growth Model and Convergence


(1) $$\begin{array}{l} Y_{it}=K_{it}^{\alpha }{\left(A_{it}L_{it}\right)}^{1-\alpha },0<\alpha <1 \end{array}$$

with


(2) $$\begin{array}{l} K_{it}=I_{i,t-1}+\left(1-\delta \right)K_{i,t-1},\\ \;I_{it}=s_{i}Y_{it} \end{array}$$

If we augment the *A*_*it*_*L*_*it*_ technology and employment in one variable, such as *HC*_*it*_ = *A*_*it*_*L*_*it*_


(3) $$\begin{array}{l} hc_{it}=Ln\left(HC_{it}\right)=\bar{h}c_{i0}+hc_{i}t+u_{it}, \end{array}$$

where Δ*u*_*it*_ is strictly stationary ergodic.

As measured by capital per effective labor units, *k*_*it*_ = *K*_*it*_/*HC*_*it*_ we have;


(4) $$\begin{array}{l} \Delta \ln\;\left(k_{it}\right)=-k_{i}-\Delta u_{it}+\ln\;\left(s_{i}k_{it}^{-\left(1-\alpha \right)}+1-\delta \right), \end{array}$$

Under firm assumption on *u*_*it*_ and assuming that 0 < α, δ < 1. Binder and Pesaran (5) indicate that *k*_*it*_ converges to time-invariant distribution for each i. As a result *y*_*it*_ = log(*Y*_*it*_/*HC*_*it*_) also converge to a steady state distribution whose evaluation is provided by,


(5) $$\begin{array}{l} y_{it}=hc_{it}+\alpha \ln\;\left(k_{it}\right), \end{array}$$

Moreover *y*_*it*_ will have the same limiting time series characteristics as *hc*_*it*_ [see detailed proof in Binder and Pesaran (5)]. In this case *y*_*it*_ has a unit root process if and only if *hc*_*it*_ has a unit root process. In this study, following these findings, we will test whether the health expenditure convergence, which means I(0) process leads to growth convergence I(0). As Binder and Pesaran (5) found that under certain assumptions, effective labor stochastic behavior directly affects the stochastic behavior of the growth variable. Therefore, this relationship may also determine the growth convergence behavior due to the stochastic behavior of health expenditure convergence. Health expenditure convergence means that the country's human capital (HC) converges to a better condition concerning the leading country or the average of the well-defined rich-income countries. Therefore, an increase or convergence in health expenditure directly means the technology improvements and/or an increase in the productivity of the human capital. Thus, we can intuitively claim that health expenditure convergence leads to similar findings with related to Binder and Pesaran (5) result where *y*_*it*_ has a unit root process only in the event of *hc*_*it*_ has a unit root process. In this empirical investigation, we will also search for the feedback effect of the growth convergence to health convergence. Most probably one convergence trigger the other converges as well.

## 4. Data and the empirical analysis

The study used the data of 22 OECD member countries for the period 1976–2020. The OECD and the EU have 11 nations as members (Austria, Belgium, Denmark, Finland, Germany, Ireland, Luxembourg, Netherlands, Portugal, Spain, and Sweden). The remaining 11 countries are members of the OECD (Australia, Canada, Iceland, Japan, Korea, New Zealand, Norway, Switzerland, Türkiye, the United Kingdom, and the United States).

The following variables were calculated to test the convergence processes between health expenditure per qualified worker and output per qualified worker^1^:


$$\begin{array}{l} Health\;expenditure\;per\;qualified\;worker:\frac{Health\;Expenditure}{h.L}\\ \;Output\;per\;qualified\;worker:\frac{GDP}{h.L} \end{array}$$

The health convergence study was conducted with linear, state-dependent non-linear, time-varying non-linear, and hybrid unit root tests. In line with these results, the Sollis (41) test achieved health convergence in all countries in the sample. The results of all tests can be seen in Table 1.

**Table 1:** Results of time series unit root tests–health expenditure per qualified worker.

	**ADF**		**KSS**		**Sollis** **(****42****)**		**EG**		**Enders and Lee**		**LNV**			**Sollis** **(****41****)**		
	**Intercept Only**	**Intercept and Trend**	**Intercept Only**	**Intercept and Trend**	**Intercept Only**	**Intercept and Trend**	**Intercept Only**	**Intercept and Trend**	**Intercept Only**	**Intercept and Trend**	**Model A**	**Model B**	**Model C**	**Model A**	**Model B**	**Model C**
Australia	−1.345	−2.759	−1.376	−2.055	0.923	3.218	1.034	4.383	−1.454	−3.535	−2.273	−3.110	−3.670	2.64553	5.090^***^	6.575^***^
Austria	−2.776^*^	−2.895	−2.860^*^	−2.438	3.988	3.550	4.258^*^	4.637	−3.123	−4.574^**^	−2.630	−3.810	−3.547	3.37806^*^	7.073^***^	6.202^***^
Belgium	−1.546	−3.195^*^	−2.034	−4.759^***^	4.032^*^	11.100^***^	1.276	5.497^*^	−3.152	−2.897	−4.343^*^	−3.368	−3.137	9.63131^***^	5.796^***^	5.079^***^
Canada	−1.297	−2.061	−1.391	−1.367	1.654	1.484	2.643	2.480	−1.194	−2.943	−1.847	−3.476	−3.956	2.18250	6.180^***^	7.633^***^
Denmark	−6.917^***^	−5.289^***^	−4.688^***^	−4.558^***^	19.815^***^	15.078^***^	25.006^***^	18.379^***^	−6.351^***^	−4.226^*^	−3.190	−4.042	−4.127	4.97079^***^	9.478^***^	8.367^***^
Finland	−0.560	−2.829	−0.795	−1.642	0.322	5.367	1.724	4.280	−1.464	−3.172	−2.564	−2.906	−3.814	3.30951	4.134^**^	7.458^***^
Germany	−0.352	−2.294	−1.132	−1.947	0.675	2.511	0.351	3.434	−1.042	−2.672	−2.940	−2.628	−4.138	4.42276^***^	3.510	8.569^****^
Iceland	−1.389	−1.093	−2.088	−1.525	3.311	2.426	0.947	1.213	−4.891^***^	−5.324^***^	−3.008	−4.853^*^	−5.559^**^	8.11764^***^	11.910^***^	15.177^***^
Ireland	−1.352	−2.151	−2.699^*^	−2.538	5.919^**^	4.442	4.362^*^	2.370	−0.749	−2.717	−3.248	−4.599	−4.322	5.19027^***^	10.907^***^	9.560^***^
Japan	−1.067	−3.508^**^	−1.162	−3.058	3.05049	4.860	5.465^**^	6.721^**^	−1.734	−4.273^*^	−2.972	−4.739^*^	−4.759	4.63492^***^	11.179^***^	11.045^***^
Korea	−2.132	−2.164	−2.232	−2.320	2.670	2.707	2.934	2.868	−2.536	−4.187^*^	−2.069	−1.925	−3.749	3.62060^*^	1.805	7.215^***^
Luxembourg	−0.967	−1.781	−0.604	−2.656	2.922	3.442	5.256^**^	1.677	−0.933	−3.297	−2.943	−4.146	−4.440	5.13121^***^	8.576^***^	10.045^***^
Netherlands	−0.737	−2.536	−1.242	−3.070	1.734	9.480^***^	3.685	4.228	−0.873	−4.399^**^	−3.427	−4.443	−3.434	5.76400^***^	9.625^***^	5.752^***^
New Zealand	−1.422	−1.096	−1.431	−0.796	1.079	0.482	1.180	0.599	−2.590	−2.569	−1.893	−2.802	−3.082	1.78781	4.061^*^	4.759^**^
Norway	−1.759	−2.221	−2.103	−2.377	3.325	2.958	1.527	2.495	−2.337	−4.946^***^	−5.195^***^	−5.238^**^	−5.446^**^	13.23172^***^	13.383^***^	14.485^***^
Portugal	−0.452	−2.827	−1.455	−2.079	1.387	2.274	0.247	4.551	−1.998	−2.729	−3.6734	−3.181	−4.003	6.63369^***^	4.934^***^	7.889^***^
Spain	−0.324	−1.762	−0.173	−2.094	0.073	2.150	0.800	1.517	−1.622	−4.240^*^	−2.879	−4.264	−4.385	4.05095^**^	9.147^***^	9.467^***^
Sweden	−1.142	−1.168	−0.811	−0.892	0.326	0.411	0.720	0.671	−1.695	−3.013	−1.083	−3.002	−3.392	1.22633	4.501^**^	5.614^***^
Switzerland	−1.632	−1.790	−2.240	−3.256^*^	5.242^**^	6.191^*^	2.314	2.629	−3.492^*^	−2.900	−2.240	−4.206	−3.786	3.10651	9.465^***^	7.700^***^
Türkiye	−0.061	−0.553	−0.159	−0.789	0.091	0.402	2.406	0.806	−0.810	−2.997	−2.255	−3.526	−2.860	2.77670	6.756^***^	4.118^*^
United Kingdom	−1.248	−1.362	−1.106	−1.479	1.547	1.100	1.231	0.934	−0.642	−2.558	−2.061	−5.249^**^	−5.482^**^	2.38588	13.474^***^	14.775^***^
United States	−1.167	−0.764	−1.511	−1.237	1.739	1.473	4.430^*^	0.428	−0.372	−2.520	−2.456	−2.899	−3.831	2.93785	4.237^**^	7.181^***^

^*^, ^**^, and ^***^ are representing 10%, 5%, and 1% significance level, respectively.

The income or output convergence study was conducted with linear, state-dependent non-linear, time-varying non-linear, and hybrid unit root tests. In line with these results, the Sollis (41) test achieved income or output in almost all countries in the sample except Spain and Ireland. The results of all tests can be seen in Table 2.

**Table 2:** Results of time series unit root tests–output per qualified worker.

	**ADF**		**KSS**		**Sollis** **(****42****)**		**EG**		**Enders and Lee**		**LNV**			**Sollis** **(****41****)**		
	**Intercept Only**	**Intercept and Trend**	**Intercept Only**	**Intercept and Trend**	**Intercept Only**	**Intercept and Trend**	**Intercept Only**	**Intercept and Trend**	**Intercept Only**	**Intercept and Trend**	**Model A**	**Model B**	**Model C**	**Model A**	**Model B**	**Model C**
Australia	−1.938	−2.209	−1.290	−1.376	0.841	0.980	2.225	3.037	−2.833	−2.853	−2.791	−3.061	−4.182	4.282^***^	5.887^***^	8.571^***^
Austria	−0.313	−2.881	−0.998	−3.170^*^	0.670	5.337	0.242	4.594	−1.396	−3.628	−3.722	−4.113	−3.718	6.988^***^	10.123^***^	6.915^***^
Belgium	−1.673	−2.611	−1.132	−1.726	1.287	1.495	1.489	3.363	−2.671	−3.245	−4.417^**^	−4.323	−3.708	9.507^***^	9.477^***^	6.809^***^
Canada	−0.427	−2.598	−0.837	−1.968	0.851	2.354	1.870	3.291	−1.550	−3.364	−3.029	−3.422	−3.817	4.966^***^	5.872^***^	7.303^***^
Denmark	−1.224	−1.642	−1.596	−1.897	1.722	2.174	0.741	1.460	−3.839^**^	−3.443	−2.144	−3.474	−3.814	2.298	6.035^***^	7.365^***^
Finland	−2.440	−2.018	−2.219	−2.699	3.276	3.594	2.906	2.006	−2.244	−4.997^***^	−2.137	−4.590	−4.496	2.522	10.287^***^	9.985^***^
Germany	−1.123	−1.616	−1.392	−1.325	0.950	0.887	0.895	1.274	−1.958	−2.831	−2.546	−2.629	−3.843	3.224	3.419	7.202^***^
Iceland	−2.071	−2.011	−1.362	−2.287	1.568	2.645	2.096	1.986	−2.890	−4.172^*^	−2.800	−4.667^*^	−4.674	4.024^**^	10.862^***^	11.223^***^
Ireland	−0.812	−1.153	−1.178	−1.349	1.136	2.059	2.939	1.535	0.061	−1.433	−1.642	−1.150	−1.903	1.740	1.832	2.06682
Japan	−0.663	−1.733	−0.786	−2.105	1.454	2.487	0.843	1.497	−1.501	−2.366	−2.950	−2.837	−2.394	4.651^***^	3.969^*^	2.81705
Korea	−1.096	−0.922	−1.429	−1.649	1.125	1.506	3.631	0.415	0.302	−3.370	−2.745	−2.722	−3.386	3.678^**^	3.653	6.570^***^
Luxembourg	−1.601	−1.349	−2.371	−1.904	4.493^*^	2.992	1.527	1.118	−3.063	−3.004	−1.116	−4.034	−3.607	3.007	8.140^***^	6.659^***^
Netherlands	−2.012	−1.777	−2.525	−2.429	3.617	3.032	2.437	1.890	−3.195	−2.669	−1.743	−3.432	−5.046	1.490	5.738^***^	12.663^***^
New Zealand	−0.170	−1.690	−0.370	−1.810	0.146	1.784	2.000	1.461	−0.691	−3.844	−2.489	−3.983	−4.393	3.175	8.439^***^	9.522^***^
Norway	−1.409	−1.010	−1.656	−1.805	1.383	1.934	0.991	0.652	−4.061^**^	−2.976	−1.445	−2.936	−6.421^***^	1.410	4.306^**^	21.049^***^
Portugal	−1.521	−1.961	−1.566	−2.058	1.196	2.290	2.712	2.402	−0.689	−3.502	−2.064	−3.872	−2.854	2.247	9.712^***^	6.627^***^
Spain	−0.125	−2.115	−0.696	−2.012	0.257	2.150	0.353	2.306	0.652	−1.354	−2.213	−1.389	−2.303	2.389	1.566	2.714
Sweden	−0.667	−2.295	−1.148	−1.546	0.740	1.735	0.483	2.713	−2.185	−3.188	−3.451	−3.189	−3.495	5.912^***^	5.227^***^	6.480^***^
Switzerland	−1.569	−2.781	−1.967	−2.319	2.606	2.631	1.639	3.865	−2.182	−2.502	−3.364	−2.589	−2.604	6.082^***^	3.268	3.309
Türkiye	−2.256	−1.885	−1.686	−1.073	2.037	1.063	4.710^*^	2.148	−2.903	−2.809	−1.782	−2.589	−3.093	2.859	4.717^**^	6.547^***^
United Kingdom	−0.006	−1.366	−0.120	−1.633	0.915	1.958	1.210	1.074	−1.569	−2.867	−2.797	−2.743	−3.256	4.431^***^	4.245^**^	5.326^***^
United States	−0.290	−2.280	−1.069	−2.626	0.568	3.367	0.387	2.539	−1.003	−4.058^*^	−3.856	−3.848	−4.278	7.375^***^	7.328^***^	9.247^***^

^*^, ^**^, and ^***^ are representing 10%, 5%, and 1% significance level, respectively.

The results from Tables 1, 2 confirm the theoretical relationship presented in section 2.3 “Binder and Pesaran (5) theoretical finding: where *y*_*it*_ has a unit root process if and only if *hc*_*it*_ has a unit root process” this theoretical finding can also be generalize to: *y*_*it*_ is a stationary process if and only if *hc*_*it*_ is a stationary process. The methodological details are given in Appendix A.

## 5. Concluding remarks

Health expenditures of countries do not only include medicine and care expenditures. Effective use of health expenditures can positively impact human capital. Furthermore, health expenditures are considered one of the critical components of human capital. In societies where health spending is ineffective, unhealthy individuals are encountered primarily. These unhealthy individuals are not expected to integrate effectively into the education system or production processes. Health expenditures, directly and indirectly, impact education and production processes in this respect.

Many factors enable the education system to function actively. Societies without healthy individuals cannot receive and provide qualified education. For the education system to function effectively, the actors in the system must be healthy. Otherwise, the process may end up in a deadlock. In this respect, the role of health expenditures is essential for the education system to work actively. Countries will see an increase in the proportion of healthy individuals in their societies if they use their health expenditures in a planned and effective manner. The increase in the rate of healthy individuals will also increase the opportunities for effectively utilizing the education system. A high-quality education received by healthy individuals within the educational system can enable them to enter the job market with high-income expectations. The income expectation of a healthy and qualified workforce in the market differs from that of an unhealthy and unqualified workforce. A healthy and qualified workforce is offered a relatively high income which, on the one hand, will raise their standard of living and, on the other hand, will enable them to consume more. One of the main components of GDP is consumption. Increasing health expenses for a healthy and qualified workforce can contribute to the country's growth by increasing GDP.

Health expenditures also affect GDP through another channel. When health expenditures increase effectively in a country, healthy individuals can receive qualified education. The quality of education healthy individuals receives shapes their expectations for wages, as mentioned above. In addition, a healthy and qualified workforce significantly impacts human capital. Countries need to invest in high human capital in order to be able to produce high-value-added products. Health expenditures can contribute directly and indirectly to increased human capital. The increase in human capital that comes with health spending can increase the country's overall productivity and open the way for producing high-value-added products. One of the critical determinants of economic growth is human capital. The role of health spending in determining human capital cannot be denied. Therefore, an increase in quality health spending in the country will lead to an increase in human capital and bring along an increase in value-added and efficient production, thereby increasing GDP.

The study of Binder and Peseran (5) starts from a similar point and shows the effect of human capital on economic growth through the degree of integration order. Similar to our study, they obtained the theoretical finding that human capital stationarity will lead to growth stationarity. In other words, a convergence in health spending will lead to a convergence in growth.

Non-linear unit root tests were applied to 22 OECD countries empirically to test the hypothesis of health expenditure and growth convergence. While the variable of health expenditure per qualified worker was used to measure health expenditure, the output variable per qualified worker was used as an indicator of growth. The analyzes covering the period 1976–2020 revealed the convergence of health expenditure per qualified worker in all countries. On the other hand, convergence for output per qualified worker was achieved in 91% of the countries. As a result, the assumption mentioned above was confirmed. Our findings are consistent with following studies (37–40).

The results reflecting the policy recommendations obtained from the analysis suggest that in order to have a stable growth path, countries need to make at least as much health expenditure as other countries. The inclusiveness and quality of health expenditures are as crucial as the quantity for the effective functioning of convergence processes. It has been observed in the study that the health expenditure data has a non-linear structure. Therefore, it should not be forgotten that incorrect results may be encountered if this situation is not considered in the analyses to be carried out regarding health expenditures. This situation can also lead to incorrect determination of health policies.

## Data availability statement

The datasets presented in this study can be found in online repositories. The names of the repository/repositories and accession number(s) can be found at: https://www.oecd.org/health/health-data.htm, https://data.oecd.org/gdp/gross-domestic-product-gdp.htm, http://www.barrolee.com/, https://www.conference-board.org/data/economydatabase/total-economy-database-productivity.

## Author contributions

All authors listed have made a substantial, direct, and intellectual contribution to the work and approved it for publication.

## Footnotes

^1^h: average education level and L: number of people employed. Health expenditures and GDP variables were accessed from the OECD database. Average training time (h) data were taken from the Barro-Lee database. Employment numbers were obtained from The Conference Board Total Economy Database.

## References

[1] Piabuo SM Tieguhong JC. Health expenditure and economic growth - a review of the literature and an analysis between the economic community for central African states (CEMAC) and selected African countries. Health Econ Rev. (2017) 7:1–13. 10.1186/s13561-017-0159-1 28593509 PMC5462666

[2] Raghupathi V Raghupathi W. Healthcare expenditure and economic performance: insights from the United States data. Front Public Heal. (2020) 8:1–15. 10.3389/fpubh.2020.00156 32478027 PMC7237575

[3] Hanushek E Wößmann L. The role of education quality in economic growth. World Bank Policy Res Work Pap. (4122) 2007:1–94. 10.1596/1813-9450-4122

[4] Alvi S Ahmed AM. Analyzing the impact of health and education on total factor productivity: a panel data approach. Indian Econ Rev. (2014) 49:109–23.26060727

[5] Binder M Pesaran MH. Stochastic growth models and their econometric implications. J Econ Growth. (1999) 4:139–83. 10.1023/A:1009802421114

[6] Pekkurnaz D. Convergence of health expenditure in OECD countries: evidence from a nonlinear asymmetric heterogeneous panel unit root test. J Rev Glob Econ. (2015) 4:76–86. 10.6000/1929-7092.2015.04.07

[7] Narayan PK. Do health expenditures “catch-up”? evidence from OECD countries. Health Econ. (2007) 16:993–1008. 10.1002/hec.1196 17238220

[8] Barro RJ Sala-i-Martin X. Economic Growth. The MIT Press (2004). Available online at: http://piketty.pse.ens.fr/files/BarroSalaIMartin2004Chap1-2.pdf%0Ahttp://www.jstor.org/stable/2937943%0Ahttps://www.journals.uchicago.edu/doi/10.1086/431086 (accessed January 27, 2023).

[9] Weddige-haaf K Kool C. Determinants of regional growth and convergence in Germany. Utr Univ Sch Econ. (2017) 17:34.

[10] Barro RJ Sala-I-Martin X. Convergence. J Polit Econ. (1992) 100:223–51.

[11] Hitiris T Nixon J. Convergence of health care expenditure in the EU countries. Appl Econ Lett. (2001) 8:223–8. 10.1080/135048501750103890

[12] Johnson P Papageorgiou C. What remains of cross-country convergence? J Econ Lit. (2020) 58:129–75. 10.1257/jel.20181207

[13] Bernard AB Durlauf SN. Convergence in international output. J Appl Econom. (1995) 10:97–108. 10.1002/jae.3950100202

[14] Jewell T Lee J Tieslau M Strazicich MC. Stationarity of health expenditures and GDP: evidence from panel unit root tests with heterogeneous structural breaks. J Health Econ. (2003) 22:313–23. 10.1016/S0167-6296(02)00122-4 12606148

[15] Dowrick S Delong JB. Globalization and convergence. In:Bordo MD Taylor AM Williamson JG, editors. Globalization in Historical Perspective. Chicago: University of Chicago Press (2003) p. 191–220.

[16] Alcalde-Unzu J Ezcurra R Pascual P. Cross-country disparities in health-care expenditure: a factor decomposition. Health Econ. (2009) 18:479–85. 10.1002/hec.1374 18613298

[17] Schmitt C Starke P. Explaining convergence of oecd welfare states: a conditional approach. J Eur Soc Policy. (2008) 21:120–35. 10.1177/0958928710395049

[18] Leiter AM Theurl E. The convergence of health care financing structures: empirical evidence from OECD-countries. Eur J Heal Econ. (2010) 13:7–18. 10.1007/s10198-010-0265-z 20640869

[19] Fallahi F. Causal relationship between energy consumption (EC) and GDP: a Markov-switching (MS) causality. Energy. (2011) 36:4165–70. 10.1016/j.energy.2011.04.027

[20] Panopoulou E Pantelidis T. Convergence in per capita health expenditures and health outcomes in the OECD countries. Appl Econ. (2012) 44:3909–20. 10.1080/00036846.2011.583222

[21] Albulescu CT. Health care expenditure in the European Union countries: new insights about the convergence process. Int J Environ Res Public Health. (2022) 19:1991. 10.3390/ijerph19041991 35206178 PMC8872178

[22] Kizilkaya F Dag M. Convergence of health expenditures in OECD countries: evidence from fourier unit root test with break. J Yasar Univ. (2021) 16:587–600. 10.19168/jyasar.880203

[23] Akarsu G Cafri R Bidirdi H. Are public-private components of health care expenditures converging among OECD Countries? evidence from a nonlinear panel unit root test. Sosyoekonomi. (2019) 27:89–112. 10.17233/sosyoekonomi.2019.03.05

[24] Lee J Tieslau M. Panel LM unit root tests with level and trend shifts. Econ Model. (2019) 80:1–10. 10.1016/j.econmod.2017.11.001

[25] Albulescu CT Oros C Tiwari AK. Is there any convergence in health expenditures across EU countries? Econ Bull. (2017) 37:2095–101.

[26] Payne JE Anderson S Lee J Cho MH. Do per capita health care expenditures converge among OECD countries? Evidence from unit root tests with level and trend-shifts. Appl Econ. (2015) 47:5600–13. 10.1080/00036846.2015.1054070

[27] Lau CKM Fung KWT Pugalis L. Is health care expenditure across Europe converging? Findings from the application of a nonlinear panel unit root test. Eurasian Bus Rev. (2014) 4:137–56. 10.1007/s40821-014-0014-9

[28] Aslan A. Convergence of per capita health care expenditures in OECD countries. Int Res J Financ Econ. (2009) 1:48–53.17238220

[29] Ucar N Omay T. Testing for unit root in nonlinear heterogeneous panels. Econ Lett. (2009) 104:5–8. 10.1016/j.econlet.2009.03.018

[30] Furuoka F Rasiah R Idris R Ziegenhain P Jacob RI Munir Q. Income convergence in the ASEAN-5 countries. Int J Bus Soc. (2018) 19:554–69.

[31] Ceylan R Abiyev V. An examination of convergence hypothesis for EU-15 countries. Int Rev Econ Financ. (2016) 45:96–105. 10.1016/j.iref.2016.05.007

[32] Yaya OOS Furuoka F Pui KL Jacob RI Ezeoke CM. Investigating Asian regional income convergence using fourier unit root test with break. Int Econ. (2020) 161:120–9. 10.1016/j.inteco.2019.11.008

[33] Lopes S Lopes AS. Income convergence with DF-Fourier tests : old evidence with a new test revisiting income convergence with DF-Fourier tests : old evidence with a new test. MPRA Pap. (2020).

[34] Holobiuc A-M. Income convergence in the european union: national and regional dimensions. Eur Financ Account J. (2020) 15:45–65. 10.18267/j.efaj.242

[35] Das RC Das U Das A. BRICS nations and income convergence: an insight from the quarterly data for 2006Q1–2017Q2. Glob Bus Rev. (2021) 22:1054–69. 10.1177/0972150918822057

[36] Alataş S. Revisiting the Solow growth model: new empirical evidence on the convergence debate. J Econ Adm Sci. (2021). 10.1108/JEAS-02-2021-0035

[37] Erçelik G. The relationship between health expenditure and economic growth in Turkey from 1980 to 2015. DergiparkOrgTr. (2018) 1:1–8.

[38] Wang F. The roles of preventive and curative health care in economic development. PLoS ONE. (2018) 13:1–12. 10.1371/journal.pone.0206808 30403708 PMC6221337

[39] Wang Y Tao C Xiong Q. Government health expenditure, economic growth, and regional development differences—analysis based on a non-parametric additive model. Front Public Heal. (2022) 10:1–18. 10.3389/fpubh.2022.925910 35899159 PMC9309553

[40] Ivankova V Gavurova B Khouri S Szabo G. Examining the economic perspective of treatable mortality: the role of health care financing and the importance for economic prosperity. Front Public Heal. (2021) 9:1–16. 10.3389/fpubh.2021.780390 34966714 PMC8710442

[41] Sollis R. Asymmetric adjustment and smooth transitions: a combination of some unit root tests. J Time Ser Anal. (2004) 25:409–17. 10.1111/j.1467-9892.2004.01911.x

[42] Sollis R. A simple unit root test against asymmetric STAR nonlinearity with an application to real exchange rates in Nordic countries. Econ Model. (2009) 26:118–25. 10.1016/j.econmod.2008.06.002

[43] Enders W Granger CWJ. Unit-root tests and asymmetric adjustment with an example using the term structure of interest rates. J Bus Econ Stat. (1998) 16:304–11.

[44] Tong H. Threshold Models in Nonlinear Time Series Models. New York, NY: Springer-Verlag (1983).

[45] Leybourne S Newbold P Vougas D. Unit roots and smooth transitions. J Time Ser Anal. (1998) 19:83–97. 10.1111/1467-9892.00078

[46] Leybourne SJ McCabe BPM Tremayne AR. Can economic time series be differenced to stationarity? J Bus Econ Stat. (1996) 14:435–46.

[47] Kapetanios G Shin Y Snell A. Testing for a unit root in the nonlinear STAR framework. J Econom. (2003) 112:359–79. 10.1016/S0304-4076(02)00202-6

[48] Omay T Emirmahmutoglu F Hasanov M. Structural break, nonlinearity and asymmetry: A re-examination of PPP proposition. Appl Econ. (2018) 50:1289–308. 10.1080/00036846.2017.1361005

[49] Enders W Lee J. A unit root test using a fourier series to approximate smooth breaks. Oxf Bull Econ Stat. (2012) 74:574–99. 10.1111/j.1468-0084.2011.00662.x35707073

