# Supplementary material for: Convergence of economic growth and health expenditures in OECD countries: Evidence from non-linear unit root tests
Source: Front Public Health. 2023 Mar 17;11:1125968. doi: 10.3389/fpubh.2023.1125968 (PMC10065192; doi:10.3389/fpubh.2023.1125968)
Supplement: Supplementary file 1 [file Data_Sheet_1.pdf]

Appendix A

Enders and Granger (1998) Test (43)

In their study Enders and Granger (43) stated that the standard unit tests previously developed focused on a symmetric adjustment process. Working from this, they have developed a unit root test that can be applied in the case of an asymmetric adjustment process towards a fixed mean. The threshold autoregressive (TAR) process discovered by Tong (44) was at the base of this particular unit root test, which can be shown as:

$$\Delta y_t = I_t \rho_1 y_{t-1} + (1 - I_t)\rho_2 y_{t-1} + \varepsilon_t$$

Here, I_t is taken as the Heaviside indicator function, and;

$$I_t = \begin{cases} 1 & if\ y_{t-1} \geq 0 \\ 0 & if\ y_{t-1} < 0 \end{cases}$$

In the equation y_t represents the long run equilibrium value and it is equal to 0. If y_{t-1} is higher than this value, adjustment can be expressed as$\rho_1 y_{t-1}$. However, if y_{t-1} is lower than this value then, now, the adjustment becomes $\rho_2 y_{t-1}$. For the process to be referred to as stationary then the following conditions must hold; $\rho_1, \rho_2 < 0$ and $\rho_1 \neq \rho_2$. If $\rho_1 = \rho_2 = 0$ condition holds, then the process can be said to have a unit root.

Leybourne, Newbold, and Vougas (LNV) (1998) Test (45)

Different than Enders and Granger (43), Leybourne, Newbold and Vougas (45) looked at the smooth transition that took place among the different regimes to show the deterministic structural change. The test's regression models are as follows:

$$Model\ A \qquad y_t = \alpha_1 + \alpha_2 S_t(\gamma, \tau) + \nu_t$$

$$Model\ B \qquad y_t = \alpha_1 + \beta_1 t + \alpha_2 S_t(\gamma, \tau) + \nu_t$$

$$Model\ C \qquad y_t = \alpha_1 + \beta_1 t + \alpha_2 S_t(\gamma, \tau) + \beta_2 t S_t(\gamma, \tau) + \nu_t$$

In the equations, the process represented by ν_t has a mean of zero and is an $I(0)$ process, while a logistic smooth transition function with a sample size of T represents $S_t(\gamma, \tau)$:

$$S_t(\gamma, \tau) = [1 + exp\{-\gamma(t - \tau T)\}]^{-1} \qquad \gamma > 0$$

As mentioned before, the structural change that exists in this modeling strategy takes place as a smooth transition instead of the instant structural break (Leybourne et al. (46)). The logistic transition function given above has extreme values of 0 and 1 and it varies between these figures. The term used to describe a model that switches between regimes gradually, being a continuous function, is "regime-switching model." The parameter γ represents the speed of this transition between two regimes that are related to the extreme values of $S_t(\gamma, \tau) = 0$ and $S_t(\gamma, \tau) = 1$, which can also be considered as the determinant for the smoothness of the transition.

The structure of each regression model shown above is unique. In Model A, y_t is stationary around the mean and it changes from the starting value α_1 to $\alpha_1 + \alpha_2$. Model B is like Model A, however, it

contains an added fixed slope component. Lastly, in Model C, intercept yet again changes to $\alpha_1 + \alpha_2$ from being just α_1. Aside from a shift in the intercept, there is also a change in the slope to $\beta_1 + \beta_2$ from β_1.

The LNV test proposes the following null and alternative hypotheses:

Null hypothesis (*H₀*) $y_t = \mu_t,\ \mu_t = \mu_{t-1} + \varepsilon_t,\ \mu_0 = \psi$

Alternative hypothesis (*Hₐ*) Model A, Model B or Model C

Null hypothesis (*H₀*) $y_t = \mu_t,\ \mu_t = \kappa + \mu_{t-1} + \varepsilon_t,\ \mu_0 = \psi$

Alternative hypothesis (*Hₐ*) Model B or Model C

The assumption is that both ε_t and υ_t are stationary process with a zero mean.

The calculation of the test statistic involves two steps:

The first step in the calculation of the test statistic involves using the Nonlinear Least Squares (NLS) method to determine the deterministic component and calculate the residuals.

$$Model\ A \qquad \hat{\upsilon}_t = y_t - \hat{\alpha}_1 - \hat{\alpha}_2 S_t(\hat{\gamma}, \hat{\tau})$$

$$Model\ B \qquad \hat{\upsilon}_t = y_t - \hat{\alpha}_1 t - \hat{\beta}_1 - \hat{\alpha}_2 S_t(\hat{\gamma}, \hat{\tau})$$

$$Model\ C \qquad \hat{\upsilon}_t = y_t - \hat{\alpha}_1 t - \hat{\beta}_1 - \hat{\alpha}_2 S_t(\hat{\gamma}, \hat{\tau}) - \hat{\beta}_2 t S_t(\hat{\gamma}, \hat{\tau})$$

The second step involves the calculation of the ADF statistic and the *t* ratio.

$$\Delta\hat{\upsilon}_t = \hat{\rho}\hat{\upsilon}_{t-1} + \sum_{i=1}^{k} \hat{\delta}_i \Delta\hat{\upsilon}_{t-i} + \hat{\eta}_t$$

Kapetanios, Shin and Snell (KSS) (2003) Test (47)

Kapetanios et al. (47) examined the potential effects of nonlinear unit root testing and presented a different approach to test for a potential unit root. They considered the presence of a unit root as the default hypothesis and the nonlinear exponential smooth transition autoregressive (ESTAR) process, which indicates stability, as the alternative hypothesis.

The test creation process started with re-parametrizing of the ESTAR model.:

$$y_t = \beta y_{t-1} + \gamma y_{t-1}[1 - \exp(-\theta y_{t-d}^2)] + \varepsilon_t$$

Which, after inserting ϕ for $\beta - 1$, becomes:

$$\Delta y_t = \phi y_{t-1} + \gamma y_{t-1}[1 - \exp(-\theta y_{t-d}^2)] + \varepsilon_t$$

The KSS test hypothesis is based on the parameter θ, which is associated with speed of the mean reversion occurs. The following hypotheses can be used to test stationary of the y_t: Null Hypothesis (H_0): $\theta = 0$ and Alternative Hypthesis (H_a) : $\theta > 0$. But, the null hypothesis cannot directly be tested

as the γ parameter is not identified under null hypothesis. The transition function was changed to the first-order Taylor approximation in Kapetanios et al.'s (47) study as a solution to this situation. The finalized extended regression model to evaluate the process' stationarity as follows:

$$\Delta y_t = \sum_{j-1}^{p} \rho_j \, \Delta y_{t-j} + \delta y_{t-j}^3 + error$$

Sollis (2009) Test (42)

Sollis (42), while conducting research, expanded upon the ESTAR framework and put a unit root test. The suggested framework allowed for the asymmetric or asymmetric nonlinear adjustment of the offered alternative hypothesis. This is denoted as the ESTAR or AESTAR model. It incorporates both an exponential and a logistic function:

$$\Delta y_t = G_t(\gamma_1, y_{t-1}) \left\{ S_t(\gamma_2, y_{t-1})\rho_1 + \left(1 - S_t(\gamma_2, y_{t-1})\right)\rho_2 \right\} y_{t-1} + \varepsilon_t$$

$$G_t(\gamma_1, y_{t-1}) = 1 - \exp\left(-\gamma_1 (y_{t-1}^2)\right) \quad \gamma_1 \geq 0$$

$$S_t(\gamma_2, y_{t-1}) = [1 + \exp(-\gamma_2 y_{t-1})]^{-1} \quad \gamma_2 \geq 0$$

Where y_{t-1} is the transition variable.

Similar to the KSS Test, the Sollis Test has an issue where a few parameters make the null hypothesis difficult to identify: $\gamma_{2,}\rho_1$ and ρ_2. In order to surmount the issue, the function was modified on two occasions: initially through the first-order Taylor expansion of the exponential function, and ultimately by broadening the logistic function. The resultant augmented regression function can be depicted as:

$$\Delta y_t = \phi_1 y_{t-1}^3 + \phi_2 y_{t-1}^4 + \sum_{i=1}^{k} \kappa_i \Delta y_{t-i} + \eta_t$$

where; H_0: $\phi_1 = \phi_2 = 0$.

SOLLIS (2004) Test (41)

The utilization of logistic functions in research has demonstrated effectiveness in detecting structural breaks, thus, it is a prevalent choice among numerous scholars to integrate them into their models. This holds true for the examination created by Omay, Emirmahmutoglu, and Hasanov (48) as well. Their examination permits both non-linear and asymmetrical adaptation towards equilibrium, in addition to enabling the occurrence of structural changes simultaneously.

The recently created examination is a fusion of the LNV Test and the SOLLIS Test. It commences with the three regression frameworks put forth by the LNV Test:

$$Model\ A \qquad y_t = \alpha_1 + \alpha_2 S_t(\gamma, \tau) + \nu_t$$

$$Model\ B \qquad y_t = \alpha_1 + \beta_1 t + \alpha_2 S_t(\gamma, \tau) + \nu_t$$

$$Model\ C \qquad y_t = \alpha_1 + \beta_1 t + \alpha_2 S_t(\gamma, \tau) + \beta_2 t S_t(\gamma, \tau) + \nu_t$$

With a sample size of T, the logistic smooth transition function expressed as follows:

$$S_t(\gamma, \tau) = [1 + exp\{-\gamma(t - \tau T)\}]^{-1} \quad \gamma > 0$$

The specific examination created by Leybourne et al. (45) employed a linear framework for the adjustment whereas; Sollis (42), previously noted, had an alternative approach. In the model proposed by Sollis (42) using the LNV-SOLLIS method, the adjustment towards equilibrium was carried out using AESTAR:

$$\Delta v_t = I_t \rho_1 v_{t-1} + (1 - I_t)\rho_2 v_{t-1} + \varepsilon_t$$

Here, I_t is taken as the Heaviside indicator function, and;

$$I_t = \begin{cases} 1 & if\ v_{t-1} \geq 0 \\ 0 & if\ v_{t-1} < 0 \end{cases}$$

In the equation yv_t represents the long run equilibrium value and it is equal to 0. If v_{t-1} is higher than this value, adjustment can be expressed as $\rho_1 v_{t-1}$. However, if v_{t-1} is lower than this value then, now, the adjustment becomes $\rho_2 v_{t-1}$. For the process to be referred to as stationary then the following conditions must hold; $\rho_1, \rho_2 < 0$ and $\rho_1 \neq \rho_2$. If $\rho_1 = \rho_2 = 0$ condition holds, then the process can be said to have a unit root.

Enders and Lee (2012) Test (49)

Using the Fourier approach has many advantages and one of them is identifying the behavior of deterministic functions of unknown forms. It is known to provide better results when compared to dummy variable methods and works even for functions that are not periodic. Enders and Lee (49) and developed a unit root test including the Fourier approach making use of its advantages.

Starting with a Dickey Fuller Test:

$$y_t = d(t) + \phi_1 y_{t-1} + \lambda t + \varepsilon_t$$

ε_t parameter refers to the stationary disturbance and has a variance σ^2. $d(t)$ represents the deterministic function in which if its functional form is known, null hypothesis can be estimated. However, if $d(t)$ is not known then ϕ_1 will equal to 1. This test developed by Enders and Lee (49) made use of the Fourier expansion, as previously mentioned, and provided an approximation for $d(t)$:

$$d(t) = \alpha_0 + \alpha sin\left(\frac{2\pi kt}{T}\right) + \beta_k cos\left(\frac{2\pi kt}{T}\right)$$

with k as the frequency and T as the number of observations. Usually when k = 1, the approximation to the model that has a structural change provides better results. The regression model used is as follows:

$$\Delta y_t = \rho y_{t-1} + c_1 + c_2 t + c_3 sin\left(\frac{2\pi kt}{T}\right) + c_4 cos\left(\frac{2\pi kt}{T}\right) + e_t$$
